# The Setting Questionnaire for the Ayahuasca Experience: Questionnaire Development and Internal Structure

**DOI:** 10.3389/fpsyg.2021.679016

**Published:** 2021-06-23

**Authors:** Alexandre Augusto de Deus Pontual, Luís Fernando Tófoli, Carlos Fernando Collares, Johannes G. Ramaekers, Clarissa Mendonça Corradi-Webster

**Affiliations:** ^1^Department of Psychology, Faculty of Philosophy, Science and Letters, University of São Paulo, Ribeirão Preto, Brazil; ^2^Interdisciplinary Cooperation for Ayahuasca Research and Outreach (ICARO), Faculty of Medical Sciences, University of Campinas, Campinas, Brazil; ^3^Department of Educational Development and Research, Faculty of Health, Medicine and Life Sciences, Maastricht University, Maastricht, Netherlands; ^4^Department of Neuropsychology and Psychopharmacology, Faculty of Psychology and Neuroscience, Maastricht University, Maastricht, Netherlands

**Keywords:** ayahuasca, setting, instrument, psychedelics, psychometrics

## Abstract

The growing interest in research on psychedelic consumption in naturalistic contexts and their possible medical and therapeutic benefits requires assessment of the relationships between the substance and the individual who consumes it (set) and its context of use (setting). This study provides a novel measurement scale for the setting of Ayahuasca consumption, the Setting Questionnaire for the Ayahuasca Experience (SQAE), and examines its psychometric properties. Construction of the scale began with a literature review, followed by interviews on 19 Ayahuasca users from different backgrounds and different consumption experience, and an online survey for quantitative data collection (*n* = 2,994). Exploratory Graph Analysis (EGA) was used to investigate the questionnaire's dimensional structure with (*n* = 1,497, half of the sample), and multidimensional item response theory (MIRT) was used to compare the fit of the theoretical dimensions with the EGA proposed dimensions (*n* = 1,497, independent other half). EGA identified six dimensions, which corresponded partially to the theorized model (Leadership, Decoration, Infrastructure, Comfort, Instruction, and Social). The MIRT comparison found that the proposed theoretical model fit significantly better than the EGA model, providing support for the former (χ^2^/df = 1,967; CFI = 0,972; TLI = 0,969; RMSEA = 0,059; WRMR = 1,087). Our findings present evidence of validity of this instrument, justifying its use for future research on the influence of the setting during the ayahuasca experience. Its findings may provide a basis for expanding the settings investigated in the use of psychedelics in general.

## Introduction

Studies with psychedelics have been steadily growing in the last two decades, with research centers in different countries investigating their effects and possible use as therapeutic tools in clinical environments (Johnson et al., 2019; Lawrence et al., 2021). There is also a growing interest in understanding how healing, self-empowerment, self-knowledge, and related processes occur with the consumption of psychedelics in naturalistic contexts (Labate, 2004; Winkelman, 2005; Luna, 2011; Gomes, 2013; Maia et al., 2020).

Beyond the pharmacological effects of the drug itself, other variables must be taken into account in addressing the total effect of this group of substances, the so-called set and setting (Hartogsohn, 2016; Haijen et al., 2018). Briefly defined, set involves variables related to the subject who is ingesting the substance—such as their personal characteristics and traits like personality and life history—and setting refers to the culture, place and situation where the consumption occurs, including decoration and objects displayed, together with what other people are present, the interpersonal relationships established among the participants, what activities are being performed and the metaphysical beliefs shared among the group (Leary and Alpert, 1962; Zinberg, 1984; MacRae, 2001; Hartogsohn, 2016). The broader beliefs regarding psychedelics are particularly relevant with the beverage ayahuasca because of its characteristic setting features that arise from its ritual origins in indigenous ritual practices from the Amazon basin and South American religious syncretism (Labate, 2004; Luna, 2011).

Ayahuasca consumption settings have different forms that stem from different traditions, cultures and their adaptations. Within each tradition, there may be different arrangements adapted for different goals. A curing ritual, for example, may be organized differently from a celebration ritual by the same group (Gomes, 2013). Nevertheless, a typical ayahuasca general setting is always composed of a spiritual leader (vegetalista, mestre, curandero, shaman, “master,” or “godfather”) who, together with their helpers, supervises the consumption of the beverage by the participants and conducts the spiritual ritual in an appropriately decorated environment (Labate, 2004). These participants, in turn, also compose the setting, typically as group rituals. Accommodations for people to sit, lay or stay on, such as chairs, cushions, hammocks or grass may also vary, but are generally present and reported as influential (Pontual et al., in revision). Singing, chanting, dancing, smoke blowing and communicating with spirits are commonly found during ayahuasca rituals, and its presentation is part of an Amerindian cosmology and its beliefs about the presence and roles undertaken by spirits, plant spirits, and ayahuasca animals (Labate, 2004).

In spite of the importance of the quality of acute psychedelic experience in determining the long-term outcomes from psychedelic experiences (Johnson et al., 2017; Roseman et al., 2018), there seems to be a lack of measurement tools available to evaluate and measure the setting and its respective impact on the experiences of ayahuasca consumers and their outcomes (Pontual et al., in revision). This statement may be broadened to the study of the setting in the field of psychedelics in general, which, although highly reliant on psychometric instruments as a way of conducting its studies (Bouso et al., 2016), seems to be lacking appropriate and modern psychometric tools to evaluate the impact of settings. Perkins et al. (2021) addressed the relationship between ayahuasca traditional settings and possible therapeutic outcomes. For this, a statistical correlation was calculated between responses on standard psychological and health questionnaires among participants of different ayahuasca denominations. Kettner et al. (2021) developed and investigated the psychometric properties of a short instrument—eight items—to assess what the authors have called Intersubjective Experience During Psychedelic Group Sessions, evaluating how participants of ayahuasca rituals related to each other during sessions and established social bonds among themselves. However, as the ayahuasca consumption setting is complex and involves other domains in addition to these, the development of more instruments is necessary.

The objective of the present study was to develop and validate a new multidimensional questionnaire with strong psychometric properties, appropriate for different ayahuasca intake contexts, that could assess the perception of the setting and be easily validated in other languages.

## Methods

### Design

An interview was first conducted with 19 ayahuasca drinkers and group leaders from different backgrounds—Santo Daime (five participants), União do Vegetal (UDV) (four participants), Shipibo tradition (two participants), Neo-Shamanic (three participants), and mixed traditions (five participants). It was opted to invite participants from different backgrounds in order to expand the perception possibilities of the ayahuasca settings, with a variety of rituals and rules. These respondents also varied in the number of experiences—from less than five experiences (three participants) to more than 500 experiences (four participants). These participants were interviewed about the setting of their consumption and how they thought these setting features related to the nature of their personal experience. Based on these interviews and on the literature on setting (Zinberg, 1984; MacRae, 2001; Labate, 2004; Hartogsohn, 2016), thematic analysis (Braun and Clarke, 2013), and scale development guidelines (Pasquali, 1998; DeVellis, 2016) were used to elaborate 33 short items (see Supplementary Material 1) in six general dimensions: Leadership, with six items about the people conducting the ceremony; Infrastructure, with seven items about the facility; Instruction, with five items about information and guidance; Social, with seven items about the other participants; Comfort, with four items about the body position; and Decoration, with four items about the place's ornamentation. To these, 15 additional descriptive questions were added about music played, activities performed, and presence of natural elements. All items were elaborated as statements that avoided idiosyncratic and culturally-specific expressions and idiomatic language that can cause problems with cross-cultural translation, helping to assure that the items would allow easy translation and cross-cultural adaptation (Hambleton et al., 2004; Vijver and Matsumoto, 2011). Items were submitted to a committee formed by five ayahuasca researchers in the field, native Portuguese speakers, —one PhD in anthropology, one PhD in biology and three PhD candidates in psychology—who judged their content and pertinence to the six general dimensions using an agreement table where items were positioned as rows and their proposed subscale as column. Items were kept in the questionnaire if they achieved a kappa score superior to 0.8. An individual video conference was then held with a sample of four ayahuasca drinkers from the lower educational level of the target demographic. These respondents were presented with the questionnaire, asked to read each item once, report if its content was simple to understand, and explain their interpretation of it to the researcher. Items that didn't achieve a perfect score on all participants were flagged to have their wording re-formulated.

Items included in the questionnaire were set up online, in Portuguese, using LimeSurvey version 1.01, on the University of Campinas server, in Brazil. The content order was randomized for each respondent, who were asked to score them on a Likert scale based on their last ayahuasca consumption: 1 - Strongly Disagree; 2 - Partially Disagree; 3 - Neither Agree nor Disagree; 4 - Partially Agree; and 5 - Strongly Agree. Invitations to participate were sent to members of ayahuasca churches, healing groups, online discussion communities and posted on social media.

Together with the setting questionnaire, there were descriptive questions and questions on demographics and on ayahuasca consumption habits and affiliations. Also added was a question about previous participation in the study to avoid repeated data. Exclusion criteria were not completing all fields/having missing data, completion of the questionnaires in <5 min—which was considered to be insufficient time—or having provided the same answer for all items—which was interpreted as invalid data. A total of 2,994 responses were considered valid and used for analysis, a sufficient number for accurate MIRT parameter estimates (Jiang et al., 2016).

### Procedures of Exploratory Graph Analysis

The responses were randomly split in two halves, for Exploratory Graph Analysis—EGA—and multidimensional item response theory—MIRT. EGA is a recently developed method from network psychometrics, that has produced comparable or better accuracy in identifying dimensions than other more common methods (e.g., principal component analysis, factor analysis, and parallel analysis; Golino and Epskamp, 2017; Golino et al., 2020). EGA consists of identification of communities of items, interpreted as possible dimensions, when they are represented in a “regularized partial correlation network” using a walktrap algorithm that computes distances *via* random walks (Pons and Latapy, 2005). The items are presented in a network made of nodes representing variables (questionnaire items) and edges representing how they are connected. The representations of these connections use penalized inverse covariances between variables to remove spuriousness. Doing so, only relevant inverse covariances remain, visually organizing items and clustering them according to their affinity to each other in a more precise way.

Correlations between items were first estimated by calculating a correlation matrix of all the variables and its inverse variance-covariance. The inverse covariance, together with a model that utilizes penalized maximum likelihood estimation to regularize it, is used to avoid overfitting. Least absolute shrinkage and selection operator (LASSO) was used as a method for regularization of the partial correlation network edges, a procedure in which a penalty [the lambda parameter (λ)] is imposed on the coefficients, an effect sufficient for some of the values to be zeroed and thus absent from the model. This absence indicated conditional independence and facilitated interpretability of the model as the communities of items tend to cluster in such a way that resemble a dimension, but without the need of loading into a latent variable as it would happen in an exploratory factor analysis. Because of the reduced number of correlations, as the regularized network becomes sparser than the non-regularized network, the clustering of items on the network becomes more self-evident (Golino and Epskamp, 2017).

The degree of regularization is determined by the variation of the Extended Bayesian Information Criterion (EBIC) (Epskamp et al., 2018). The parameter adjusted through EBIC, the gamma hyperparameter (γ), determines the final number of edges that are retained in the network. This is useful to avoid overfitting of the model. The final design of the partial item correlation network is determined by a pairwise Markov random field model, more specifically the Gaussian Graphical Model (GGM). This model generates an undirected network based on the assumption that edges indicate a full conditional association between the two given nodes after conditioning on all other nodes in the network. The Fruchterman-Reingold algorithm is used to iteratively compute the optimal placement of nodes, resulting with the most central nodes placed centralized, least central nodes in the periphery. For the EGA calculations, the R software version 4.0.2 was used (R Core Team, 2020) together with the packages lavaan, semPlot, psych, ega, igraph, qgraph were applied (Csardi and Nepusz, 2006; Epskamp et al., 2012; Rosseel, 2012; Epskamp, 2015; Golino and Epskamp, 2017; Revelle, 2017).

### Procedures of Multidimensional Item Response Theory Analysis

The second half of the sample was used for a confirmatory cross-validation of the original proposed theoretical factors and the solution using the communities of items found in the EGA. A multidimensional item response theory approach was used for this analysis, namely Samejima graded response model, with parameter estimation performed by the weighted least squares means and variances adjusted (WLSMV). This approach is similar to a confirmatory factor analysis, but instead of assuming one linear regression for each item loaded on a latent variable, it estimates one function for each Likert-scale category of each item. This approach was chosen instead of a classic confirmatory factor analysis because of the multivariate non-normality of the data, suggesting that the observed variables should be treated as categorical-ordinal, something enabled by item response theory, instead of the linear approach used in classical confirmatory factor analysis (Samejima, 1997; Osteen, 2010).

The degrees to which the observed data followed the theoretical model, as well as the model suggested by the item communities found in the EGA, were evaluated by several goodness-of-fit indices: chi-square divided by degrees of freedom (χ^2^/df), Comparative Fit Index (CFI), Tucker-Lewis Index (TLI), Weighted Root Mean Square Residual (WRMR), and Root Mean Square Error of Approximation (RMSEA). The model was considered to have a good fit with values of χ^2^/df <5 (Ullman and Bentler, 2003), CFI higher than 0.95, TLI higher than 0.95 (Hu and Bentler, 1999), WRMR lower than 1.5 (Hu and Bentler, 1999), and RMSEA lower than 0.05 (Browne and Cudeck, 1992).

The quality of each item was evaluated in terms of discrimination, R-squared and residual variances. These indicators were not used as threshold to determine item exclusions, but any items presenting a low discrimination, a low R-squared coefficient and a high residual variance were scrutinized for its appropriateness and content validity.

### Reliability

Cronbach's alpha coefficients were calculated for the full questionnaire and its six subscales. Coefficients above 0.7 were considered acceptable (Streiner et al., 2015). In addition to the alpha, Gutmann and McDonalds coefficients, we have also calculated individual reliability estimates based on the individual estimates for the standard error of measurement from the Samejima graded response model.

### Ethics

The study was approved by the Research Ethics Committee of the University of São Paulo and an Informed Consent Form was presented to all participants (Authorization number 64130517.8.0000.5407).

## Results

During the development phase, two items from the 33 elaborated—items number 02 and 28—, didn't achieve a satisfactory agreement between all members of the committee formed by five researchers on the field, achieving a kappa score of <0.8 and were flagged to be removed from the questionnaire. Among the semantic judges formed from ayahuasca drinkers of the lower educational demographic, only one item was not fully comprehensible to a respondent after first reading and was reworded—item number 12. All items are listed in Table 1, including removed items.

**Table 1:** Item statistics.

				**Multidimensional IRT analysis**	
**Item**	**Responses mean**	**sd**	**Item-rest correlation**	*a* parameter^*^	***r*^2^**
L1 Confiei todas minhas preocupações ao grupo de apoio do ritual.	4.174	1.229	0.307	0.887	0.874
L2 Me senti desamparado e tendo que cuidar de mim^−^	1.313	0.787	0.473	0.884	0.871
L3 Quem teve necessidade foi prontamente atendido.	4.797	0.603	0.443	0.884	0.872
L4 A liderança do ritual me transmitiu segurança.	4.825	0.566	0.557	0.882	0.871
L5 Os organizadores se mostraram inexperientes^−^	1.286	0.874	0.327	0.887	0.873
L6 Tive dúvidas quanto à capacidade dos organizadores em lidar com possíveis intercorrências^−^	1.485	1.059	0.546	0.882	0.868
D0 O lugar tinha características em comum com outros ambientes que frequento no dia-a-dia.^#^	3.005	1.443	0.160	0.890	0.880
D1 Para o meu gosto, a decoração estava adequada.	4.600	0.889	0.407	0.885	0.872
D2 Eu mudaria algum objeto ou imagem da decoração^−^	1.616	1.109	0.390	0.886	0.872
D3 Certos componentes do ritual não estavam de acordo com a minha espiritualidade pessoal^−^	1.632	1.119	0.477	0.884	0.870
C1 Minha posição física foi confortável durante o ritual.	4.426	0.968	0.442	0.885	0.871
C2 Gostaria de ter ficado em outra posição durante o ritual^−^	1.885	1.254	0.456	0.885	0.870
C3 O lugar em que eu estava sentado/deitado me incomodava^−^	1.576	1.056	0.510	0.884	0.869
C4 Senti falta de um apoio para a coluna, cabeça ou braços^−^	1.870	1.309	0.452	0.885	0.871
I0 A cerimônia foi realizada em um espaço suficientemente aberto.^#^	4.439	1.072	0.251	0.888	0.875
I1 Me senti confinado^−^	1.330	0.908	0.403	0.885	0.872
I2 Me preocupei com a circulação do ar naquele local^−^	1.464	1.062	0.342	0.887	0.873
I3 Achei o banheiro inadequado^−^	1.551	1.103	0.378	0.886	0.872
I4 Havia locais acessíveis para fazer minhas necessidades.	4.809	0.592	0.356	0.886	0.873
I5 Me preocupei com a falta de saídas de emergências ou algo relacionado à segurança^−^	1.269	0.813	0.426	0.885	0.871
I6 Havia um local adequado para vomitar.	4.691	0.787	0.330	0.887	0.873
G0 Aconteceram eventos que me pegaram de surpresa^−^^,^^#^	2.352	1.513	0.306	0.887	0.876
G1 O ritual ocorreu conforme o esperado.	4.621	0.774	0.405	0.885	0.872
G2 Do começo ao fim, o ritual pareceu sob controle.	4.692	0.755	0.384	0.886	0.872
G3 Fui previamente instruído quanto a todo o ritual.	4.733	0.736	0.418	0.885	0.872
G4 Houve momentos em que senti falta de instruções^−^	1.407	0.960	0.504	0.883	0.870
S0 Os outros participantes se assemelham aos meus amigos.^#^	3.891	1.161	0.338	0.887	0.873
S00 Olhar para as outras pessoas me incomodava^−^^,^^#^	1.777	1.144	0.325	0.887	0.874
S1 Os outros participantes pareciam estar bem.	4.346	0.909	0.418	0.885	0.871
S2 Tenho características em comum com aquele grupo de pessoas.	4.398	0.841	0.459	0.884	0.871
S3 Me considerei diferente dos outros participantes^−^	1.861	1.159	0.443	0.885	0.871
S4 Me senti entre iguais naquele grupo.	4.531	0.839	0.529	0.883	0.870
S5 Qual das imagens melhor representa como você se sentiu em relação ao grupo durante a sessão/cerimônia? 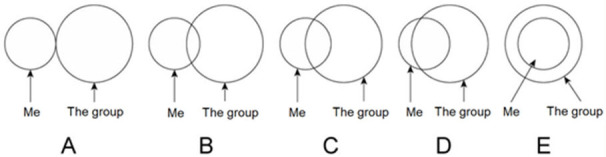	4.294	1.106	0.553	0.882	0.868

− *Reverse-scaled item*.

# *Removed item*.

* *All estimates were significant with a p-value < 0.001*.

For the investigation of evidence of validity of the questionnaire, data from 3,472 participants was collected. After application of the exclusion criteria, a total of 2,994 responses were considered valid and used for analysis. Table 2 reports their demographics. Gender and age groups were well-distributed among the sample, with the largest group being 31–40 years old (*n* = 934). The majority of the participants had completed a major or a professional education degree (81.4%). And it is observed that regarding the number of experiences, the most frequent responses (51.2%) were grouped in the highest classification, more than 100 experiences with ayahuasca. Participants who had their last experience in a União do Vegetal ritual were the largest group of respondents (53.6%).

**Table 2:** Demographic characteristics of the total sample.

		***n* = 2,994**	**Percent**
Gender	Male	1,248	48.8
	Female	1,286	50.2
	Other / Prefer not to answer	25	1.0
	Missing^a^	435	14.5
Age	18–23 years old	395	13.2
	24–30 years old	665	22.2
	31–40 years old	934	31.2
	41–60 years old	880	29.4
	+60 years old	120	4.0
Highest level of	Basic Education	60	2.0
education	Middle Education / High School	497	16.6
	Major / Professional	2,437	81.4
Number of	One	126	4.2
ayahuasca	Less than five	210	7.0
experiences	Between 5 and 20	452	15.1
	Between 20 and 100	673	22.5
	More than 100	1,533	51.2
Ritual denomination	Barquinha	13	0.4
	Neo-Shamanic	594	19.8
	Traditional Indigenous	63	2.1
	Santo Daime	604	20.2
	União do Vegetal	1,605	53.6
	Other	74	2.5
	Don't know	41	1.4

a *The first online version didn't have a question about gender*.

### SQAE Dimensions

**Figure 1:**
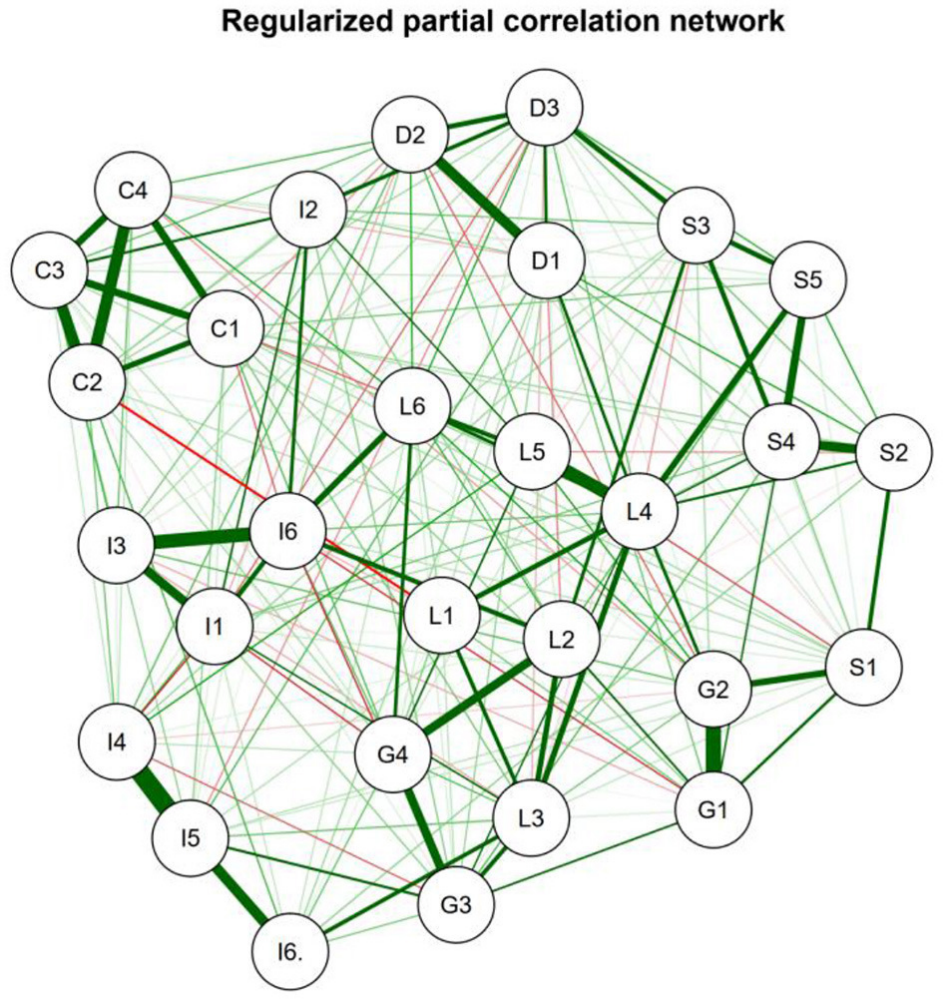
Regularized partial correlation network of the SQAE items.

**Figure 2:**
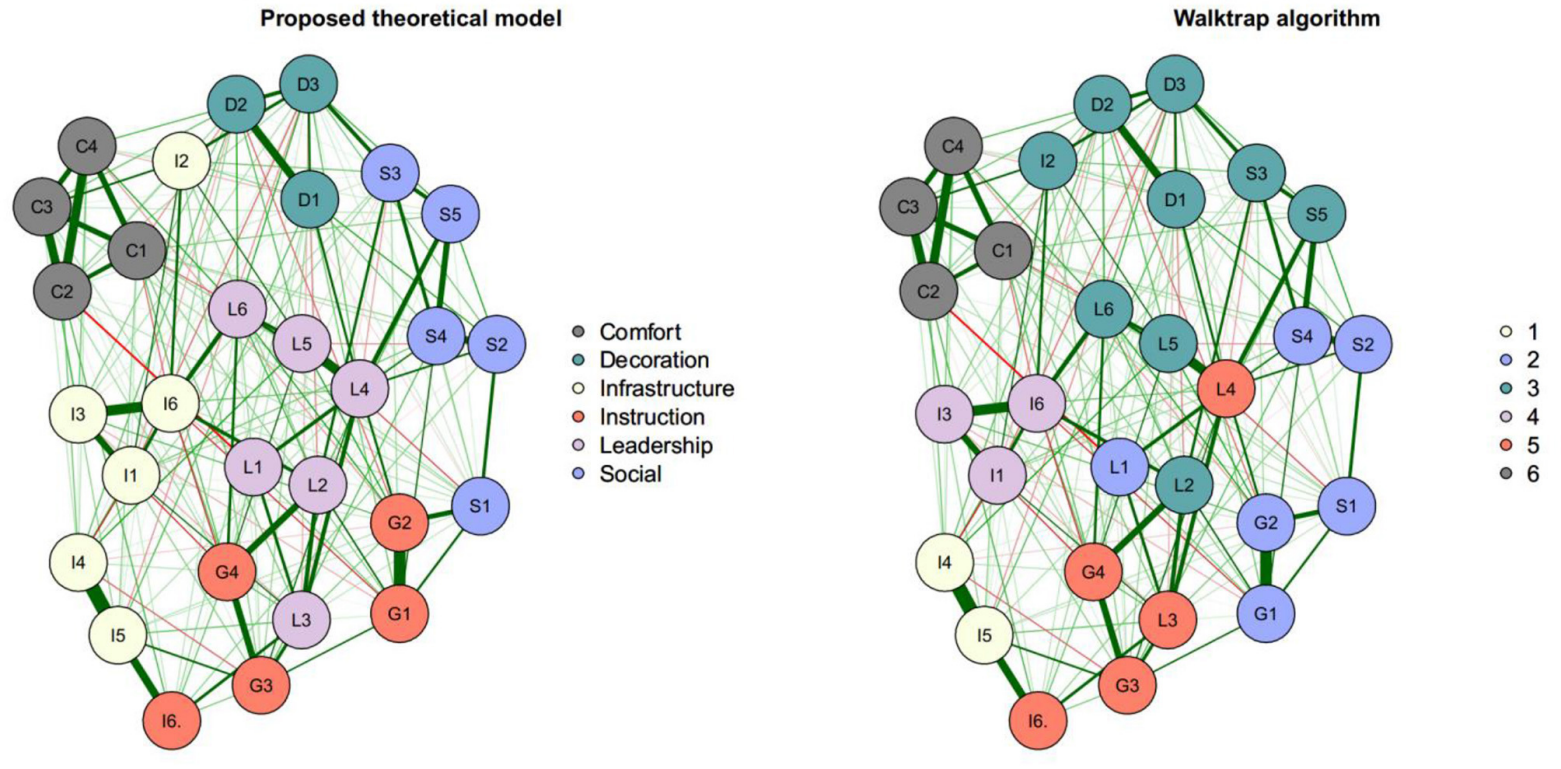
Regularized partial correlation networks: theoretical model and Walktrap. Left: proposed theoretical model. Right: EGA results using the walktrap algorithm.

EGA was conducted to explore the factor structure underlying the SQAE with half of the valid responses (*n* = 1,497). The result of the EGA analyses revealed six communities of items, which were, in overall terms, compatible with the six-factor theoretical model (Figure 1). The constructs according to the proposed theoretical model are: Social (S1 to S5), Leadership (L1 to L6), Decoration (D1 to D3), Comfort (C1 to C4), Infrastructure (I1 to I6), and Instruction (G1 to G5) (Figure 2). Figure 1 depicts a regularized partial correlation network between items—nodes—and their regularized partial correlations—edges. The thickness of the edge is the degree of correlation, with positive correlations depicted as green, and negative as red. A strong correlation also brings their respective items closer. It is possible to observe the proximity of the items from the same proposed theoretical construct. The Leadership dimension took on a centralized position, having more interconnection with other dimensions, especially with Instruction; in contrast, Comfort assumed a marginal position, with less correlation with other dimensions. Infrastructure item I2 wasn't positioned well and was removed after it also presented a poor adjustment on the MIRT.

Figure 2 shows the original proposed theoretical model and the model proposed by the walktrap algorithm. Nodes of the same color indicate common dimensionality, with the color of the edges representing positive (green) or negative (red) correlation, and their thickness representing their strength. The number of dimensions proposed to be kept by it is befitting with the proposed theoretical model, but it varies on the sixth dimension, sectioning the Infrastructure dimension in two and grouping together Leadership with Instruction. It also disagrees on some items, specially centralized items (Figure 2).

Taking the walktrap at face value for a model, both models would present fit indices in multidimensional item response theory analysis used for confirmatory purposes (*n* = 1,497) that suggest good fit, but with the theoretical model presenting superior results in all indices (Table 3), with χ^2^/df = 2.387; CFI = 0.963; TLI = 0.958; SRMR = 0.052; RMSEA [HI95%] = 0.030; Diff. test = 367.466 (15).

**Table 3:** Goodness-of-fit indices for the item response theory analyses according to the tested models.

	**Fit indices**					
	**χ^**2**^/df**	**CFI**	**TLI**	**SRMR**	**RMSEA**	**Diff. test**
Unidimensional	4.897	0.890	0.881	0.071	0.051	-
Multidimensional, as suggested by EGA	3.398	0.935	0.927	0.060	0.040	496.503(16)^*^
Multidimensional, according to theoretical framework	2.387	0.963	0.958	0.052	0.030	367.466(15)^*^

* *Chi-square difference testing with unidimensional model was significant with p < 0.001*.

The internal consistency of the SQAE and its subscales are described in Table 4. The SQAE presented acceptable reliability coefficients, with the Social, Comfort and Leader individual subscales also presenting acceptable internal consistency indices, and Infrastructure, Decoration and Instruction presenting scores below the 0.7 threshold.

**Table 4:** Scale reliability statistics.

	**Mean**	**Sd**	**McDonald's ω**	**Cronbach's α**	**Gutmann's λ6**	**Greatest lower bound**	**Average interitem correlation**
SQAE	3.097	1.620	0.862	0.860	0.875	0.905	0.192
Social	3.620	1.260	0.712	0.704	0.674	0.745	0.334
Leadership	2.971	1.878	0.622	0.613	0.619	0.648	0.240
Infrastructure	2.465	1.776	0.542	0.536	0.515	0.585	0.169
Decoration	2.562	1.755	0.552	0.547	0.450	0.552	0.289
Instruction	3.879	1.723	0.559	0.559	0.502	0.598	0.246
Comfort	2.364	1.457	0.761	0.752	0.703	0.771	0.439

Table 5 presents a correlation matrix between the subscales of the SQAE. With exception of Instruction and Leadership, that correlates highly−0.941—, statistically suggesting a possible common dimensionality, almost all correlations presented a good coefficient, between 0.623 and 0.840.

**Table 5:** Correlation matrix of the subscales.

	**Leadership**	**Decoration**	**Comfort**	**Infrastructure**	**Instruction**	**Social**
Leadership						
Decoration	0.766					
Comfort	0.623	0.630				
Infrastructure	0.840	0.799	0.637			
Instruction	0.941	0.800	0.649	0.830		
Social	0.827	0.766	0.628	0.645	0.874	

*All estimates were significant with a two-tailed p-value < 0.001.

**Figure 3:**
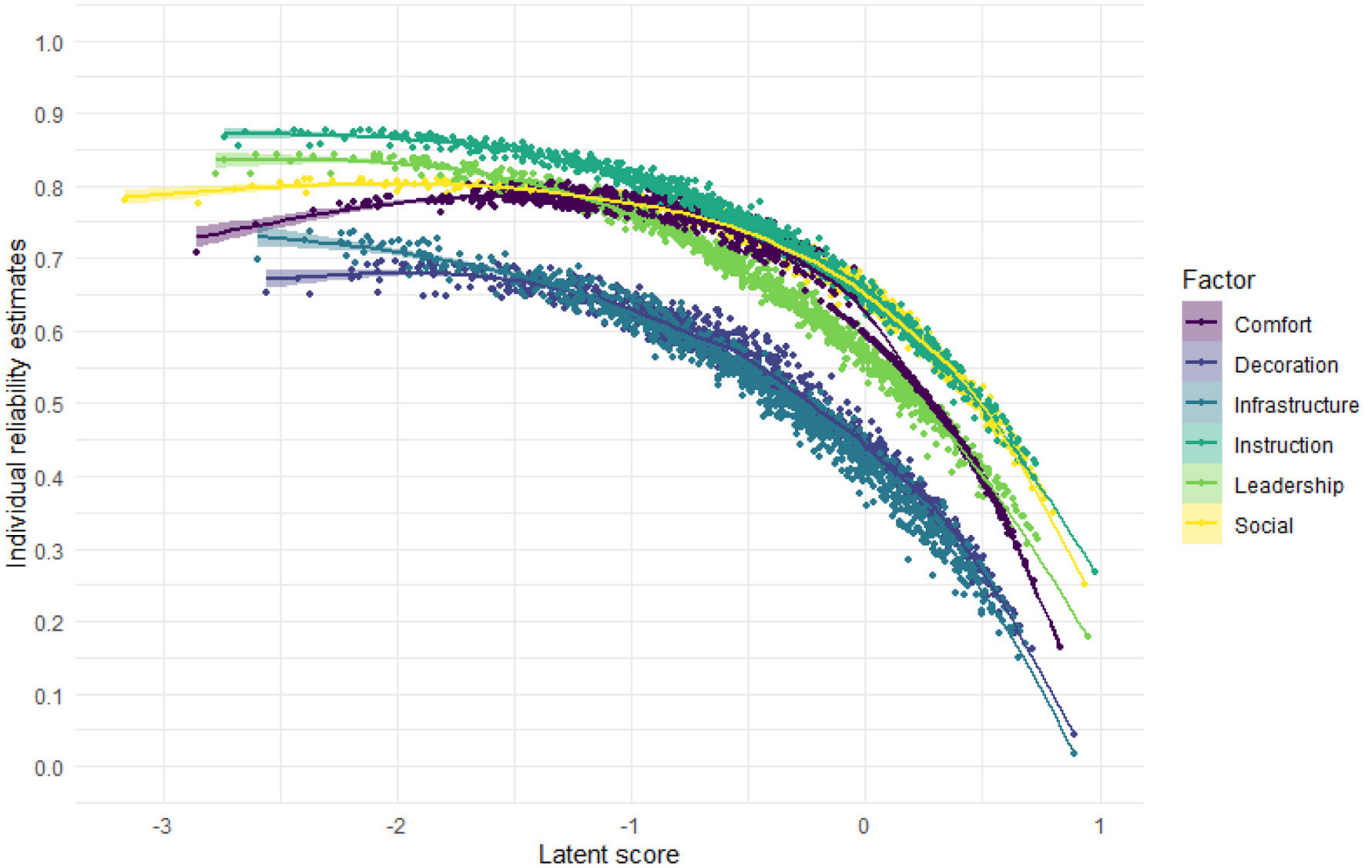
Individual reliability estimates according to the theoretical dimensions from the multidimensional item response theory analysis of the SQAE.

Figure 3 depicts individual reliability estimates for each individual participant in the study according to the proposed theoretical dimensions. Individual responses that tended to weight frequently on multiple items' extreme (1 - Strongly Disagree on reversed items or 5 - Strongly Agree on direct items) showed decreased reliability in comparison to more moderated responses.

## Discussion

This study has demonstrated the processes used for the development of the Setting Questionnaire for the Ayahuasca Experience (SQAE) as a tool to help researchers to investigate the setting component of ritualistic ayahuasca consumption, and the properties of the data collected on a large Brazilian sample. The study proceeded further to obtain evidence of validity for the SQAE based on its internal structure using exploratory graph analysis and multidimensional item response theory analysis. The semantic, expert and statistical analyses revealed that most items developed for the SQAE performed as expected, in alignment to the theoretical framework used for the SQAE construction. The study also demonstrated an acceptable of reliability for the overall instrument and for two of its subscales—Social and Comfort—using internal consistency coefficients calculated for the sample. However, conditional reliability curves calculated using the individual standard errors of measurement from the multidimensional item response theory analysis demonstrated that diversity tends to decrease in those candidates with a higher level of overall endorsement in the items of all subscales, homogenizing the responses and decreasing the reliability of the subscales with this data.

Significantly, both gender and age differences were well-represented, since the perceptions of setting may be different between genders or age groups. Participants from the highest education groups were over-represented, likely because the data collection was online. The total sample included people with many experiences as well as those little and medium experiences, guaranteeing a view of the setting from a range of experiences.

In comparison with other protocols used for factor extraction (e.g., Principal Component Analyses, Kaiser Rule, and Varimax rotation), EGA and MIRT can be considered modern and rigorous statistical analysis, and to achieve these results with a large database can be considered a positive evidence of validity. Some items and subscales indeed presented better psychometric indicators than others, but all items included were justified by theoretical thresholds or theoretical arguments. It is also possible to infer from the items' means (Table 1) that the sentences are postulated in a format that facilitates responses too close to extremes (1 - Strongly Disagree or 5 - Strongly Agree), leading to a reduced individual reliability (Image 3) and consequently lowering subscales reliability (Table 4). This frequency of extreme responses is not uncommon in studies that report responses' means with instruments in the field (Bouso et al., 2016) and could be scrutinized and improved in future versions of the questionnaire, wording items on low reliability subscales in a way that promote more heterogeneous levels of endorsement.

To start a psychometric investigation of a broad theme such as the setting is a complex venture, and many decisions had to be made. During the interviews, some influential aspects of the setting were brought to light by different participants with distinct points of views. For some participants, for example, leaders have responsibility to assure participants are fully informed a priori about what to expect of the ritual and how to behave in different situations, but for others good leadership has more to do with a spiritual and energetic issue rather than formalities, which should be regarded as different topic. These two subscales, Leadership and Instruction, were the closest in correlation among all six scales, something that can clearly be seen with the EGA and in the correlation matrix between the subscales. Another hard decision that had to be made was the exclusion of the items D0—“the place had characteristics in common with other environments that I frequent in everyday life” and S0—“the other participants are similar to my friends”—from the subscales Decoration and Social, respectively. Although these items were introduced based on interview reports, they were removed because they strongly correlated with each other, creating an undesirable new dimension “Familiarity,” that was opted out for not being contemplated by the initial reviewed literature and the theory adopted.

Although dealing with a new endeavor and not having other similar instruments to compare, we believe that the SQAE can be well-paired with other instruments that have been recently developed to be used in psychedelic studies, such as the Emotional Breakthrough Inventory (EBI) (Roseman et al., 2019), Challenging Experience Questionnaire (CEQ) (Barrett et al., 2016), recent versions of the Mystical Experience Questionnaire (MEQ) (Barrett et al., 2015; Schenberg et al., 2017), and 5d-ASC (Studerus et al., 2010). If the broadly accepted assumptions of the effects of set and setting are accurate, a positive measurement of the setting should positively correlate with a less challenging experience, and promote more mystical experience and higher levels on Emotional Breakthroughs. If the scores on these instruments are intercorrelated, it shouldn't be interpreted as redundant information: on the contrary, it should be considered as valuable predictive information for the psychedelic experience and a guide on where to manipulate to improve the chances to achieve the desirable outcome with the psychedelic use. Right now, the combined EBI, MEQ, and CEQ model was able to predict close to 20% of the variance in well-being changes after a psychedelic experience (Roseman et al., 2019), and we believe that the investigation of the setting with the SQAE can be the next step to improve this number.

We also believe that bringing modern and avant-garde techniques—such as EGA, network analysis and IRT—to psychedelic psychometrics can have a positive impact, making these assessments more comparable with other areas of the field that are also using modern techniques and high standard techniques. In principle, one interesting direction for future research would be the use of cognitive diagnostic modeling to further investigate the internal structure of the SQAE, given the apparent within-item multidimensionality of some of the items.

The elaboration and investigation of the validity of the SQAE is the beginning of a long journey that will need use, improvements and future adaptations to extend its capacity to measure the various dimensions of setting and predicting their influence on the psychedelic experience. For this, more data and broad-use are needed. This was the premise of this work, so both in the elaboration of the items and in the statistical analysis, many precautions were taken to assure each step, testing their validity and realiability, to provide foundations that can accommodate changes and future improvements.

## Data Availability Statement

The raw data supporting the conclusions of this article will be made available by the authors, without undue reservation.

## Ethics Statement

The studies involving human participants were reviewed and approved by Research Ethics Committee of the University of São Paulo. The patients/participants provided their written informed consent to participate in this study.

## Author Contributions

AP, CC-W, and LT devised the project and designed the study. AP and CC developed the theory and performed the computations. JR verified the analytical methods. AP took the lead in writing the manuscript. All authors provided critical feedback and helped shape the research, analysis, and manuscript.

## Conflict of Interest

The authors declare that the research was conducted in the absence of any commercial or financial relationships that could be construed as a potential conflict of interest.

## References

[B1] Barrett F. S. Bradstreet M. P. Leoutsakos J. S. Johnson M. W. Griffiths R. R. (2016). The challenging experience questionnaire: characterization of challenging experiences with psilocybin mushrooms. J. Psychopharmacol. 30, 1279–1295. 10.1177/0269881116678781 27856683 PMC5549781

[B2] Barrett F. S. Johnson M. W. Griffiths R. R. (2015). Validation of the revised mystical experience questionnaire in experimental sessions with psilocybin. J. Psychopharmacol. 29, 1182–1190. 10.1177/0269881115609019 26442957 PMC5203697

[B3] Bouso J. C. Pedrero-Pérez E. J. Gandy S. Alcázar-Córcoles M. Á. (2016). Measuring the subjective: revisiting the psychometric properties of three rating scales that assess the acute effects of hallucinogens. Hum. Psychopharmacol. 2016, 356–372. 10.1002/hup.2545 27470427

[B4] Braun V. Clarke V. (2013). Successful Qualitative Research: A Practical Guide for Beginners. London: Sage.

[B5] Browne M. W. Cudeck R. (1992). Alternative ways of assessing model fit. Sociol. Methods Res. 21, 230–258. 10.1177/0049124192021002005

[B6] Csardi G. Nepusz T. (2006). The Igraph software package for complex network research. Inter J. Complex Syst. 1695, 1–9.

[B7] DeVellis R. F. (2016). Scale Development: Theory and Applications, Vol. 26. Thousand Oaks, CA: Sage publications.

[B8] Epskamp S. (2015). SemPlot: unified visualizations of structural equation models. Struct. Equat. Model. Multidiscipl. J. 22, 474–483. 10.1080/10705511.2014.937847

[B9] Epskamp S. Borsboom D. Fried E. I. (2018). Estimating psychological networks and their accuracy: a tutorial paper. Behav. Res. Methods 50, 195–212. 10.3758/s13428-017-0862-1 28342071 PMC5809547

[B10] Epskamp S. Cramer A. O. J. Waldorp L. J. Schmittmann V. D. Borsboom D. (2012). Qgraph: network visualizations of relationships in psychometric data. J. Statist. Softw. 48, 1–18. 10.18637/jss.v048.i04

[B11] Golino H. F. Epskamp S. (2017). Exploratory graph analysis: a new approach for estimating the number of dimensions in psychological research. PLoS ONE 12:e0174035. 10.1371/journal.pone.0174035 28594839 PMC5465941

[B12] Golino H. F. Shi D. Christensen A. P. Garrido L. E. Nieto M. D. Sadana R.. (2020). Investigating the performance of exploratory graph analysis and traditional techniques to identify the number of latent factors: a simulation and tutorial. Psychol. Method. 2020:255. 10.1037/met0000255 32191105 PMC7244378

[B13] Gomes B. R. (2013). Ayahuasca e Recuperação de Pessoas Em Situação de Rua [Ayahuasca and Recovery of People on the Streets]. Saúde & Transformação Social/Health Social Change 4, 91–98. Available online at: http://stat.necat.incubadora.ufsc.br/index.php/saudeetransformacao/article/view/2247/2635

[B14] Haijen E. C. H. M. Kaelen M. Roseman L. Timmermann C. Kettner H. Russ S.. (2018). Predicting responses to psychedelics: a prospective study. Front. Pharmacol. 9:897. 10.3389/fphar.2018.00897 30450045 PMC6225734

[B15] Hambleton R. K. Merenda P. F. Spielberger C. D. (2004). Adapting Educational and Psychological Tests for Cross-Cultural Assessment. Mahwah, NJ: Psychology Press. 10.4324/9781410611758

[B16] Hartogsohn I. (2016). Set and setting, psychedelics and the placebo response: an extra-pharmacological perspective on psychopharmacology. J. Psychopharmacol. 30, 1259–1267. 10.1177/0269881116677852 27852960

[B17] Hu L. Bentler P. M. (1999). Cutoff criteria for fit indexes in covariance structure analysis: conventional criteria versus new alternatives. Struct. Equat. Model. Multidiscipl. J. 6, 1–55. 10.1080/10705519909540118

[B18] Jiang S. Wang C. Weiss D. J. (2016). Sample size requirements for estimation of item parameters in the multidimensional graded response model. Front. Psychol. 7:109. 10.3389/fpsyg.2016.00109 26903916 PMC4746434

[B19] Johnson M. W. Garcia-Romeu A. Griffiths R. R. (2017). Long-term follow-up of psilocybin-facilitated smoking cessation. Am. J. Drug Alcohol Abuse 43, 55–60. 10.3109/00952990.2016.1170135 27441452 PMC5641975

[B20] Johnson M. W. Hendricks P. S. Barrett F. S. Griffiths R. R. (2019). Classic psychedelics: an integrative review of epidemiology, therapeutics, mystical experience, and brain network function. Pharmacol. Therapeut. 197:83–102. 10.1016/j.pharmthera.2018.11.010 30521880

[B21] Kettner H. Rosas F. E. Timmermann C. Kärtner L. Carhart-Harris R. L. Roseman L. (2021). Psychedelic communitas: intersubjective experience during psychedelic group sessions predicts enduring changes in psychological well-being and social connectedness. Front. Pharmacol. 12:234. 10.3389/fphar.2021.623985 33995022 PMC8114773

[B22] Labate B. C. (2004). A Reinvenção Do Uso Da Ayahuasca Nos Centros Urbanos. Campinas: Editora Mercado de Letras.

[B23] Lawrence D. W. Sharma B. Griffiths R. R. Carhart-Harris R. (2021). Trends in the top-cited articles on classic psychedelics. J. Psychoactive Drug. 2021, 1–16. 10.1080/02791072.2021.1874573 33535907

[B24] Leary T. Alpert R. (1962). Foreword. Joyous Cosmol. Advent. Chem. Consciousn. 1962, 1–3.

[B25] Luna L. E. (2011). Indigenous and mestizo use of ayahuasca: an overview. Ethnopharmacol. Ayahuasca 2, 1–21.

[B26] MacRae E. (2001). Antropologia: aspectos sociais, culturais e ritualísticos. In: Seibel SD Toscano A Jr. editors. Dependência de drogas (São Paulo: Atheneu), 25–34.

[B27] Maia L. O. Daldegan-Bueno D. Tófoli L. F. (2020). The ritual use of ayahuasca during treatment of severe physical illnesses: a qualitative study. J. Psychoactive Drug. 2020, 1–11. 10.1080/02791072.2020.1854399 33287690

[B28] Osteen P. (2010). An introduction to using multidimensional item response theory to assess latent factor structures. J. Soc. Soc. Work Res. 2, 66–82. 10.5243/jsswr.2010.6

[B29] Pasquali L. (1998). Princípios de Elaboração de Escalas Psicológicas. Revista de Psiquiatria Clínica 25, 206–213.

[B30] Perkins D. Schubert V. Simonová H. Tófoli L. F. Bouso J. C. Horák M.. (2021). Influence of context and setting on the mental health and well-being outcomes of ayahuasca drinkers: results of a large international survey. Front. Pharmacol. 12:469. 10.3389/fphar.2021.623979 33967757 PMC8097729

[B31] Pons P. Latapy M. (2005). Computing communities in large networks using random walks, in International Symposium on Computer and Information Sciences (Berlin, Heidelberg: Springer), 284–293. 10.1007/11569596_31

[B32] Pontual A. A. D. Corradi-Webster C. Daldegan-Bueno D. Senhorini H. Tófoli L. F. (in revision). Systematic review of psychometric instruments used in research with psychedelics. 10.1080/02791072.2022.2079108 35616606

[B33] Pontual A. A. D. Corradi-Webster C. Ramaekers J. Tófoli L. F. (in revision). The influential components of the setting in the ayahuasca experience.

[B34] R Core Team (2020). R: A language and environment for statistical computing. Vienna: R Foundation for Statistical Computing. Available online at: https://www.R-project.org/

[B35] Revelle W. R. (2017). Psych: Procedures for Personality and Psychological Research.

[B36] Roseman L. Haijen E. Idialu-Ikato K. Kaelen M. Watts R. Carhart-Harris R. (2019). Emotional breakthrough and psychedelics: validation of the emotional breakthrough inventory. J. Psychopharmacol. 33, 1076–1087. 10.1177/0269881119855974 31294673

[B37] Roseman L. Nutt D. J. Carhart-Harris R. L. (2018). Quality of acute psychedelic experience predicts therapeutic efficacy of psilocybin for treatment-resistant depression. Front. Pharmacol. 8:974. 10.3389/fphar.2017.00974 29387009 PMC5776504

[B38] Rosseel Y. (2012). Lavaan: an R package for structural equation modeling and more. Version 0.5–12 (BETA). J. Statist. Softw. 48, 1–36. 10.18637/jss.v048.i02

[B39] Samejima F. (1997). Graded response model, in Handbook of Modern Item Response Theory, eds van der Linden W. J. Hambleton R. K. (New York, NY: Springer Science & Business Media), 85–100. 10.1007/978-1-4757-2691-6_5

[B40] Schenberg E. E. Tofoli L. F. Rezinovsky D. Silveira D. X. (2017). Translation and cultural adaptation of the states of consciousness questionnaire (SOCQ) and statistical validation of the mystical experience questionnaire (MEQ30) in Brazilian Portuguese. Archiv. Clin. Psychiatry 44, 1–5. 10.1590/0101-60830000000105

[B41] Streiner D. L. Norman G. R. Cairney J. (2015). Health Measurement Scales: A Practical Guide to Their Development and Use. Oxford, MS: Oxford University Press. 10.1093/med/9780199685219.001.0001

[B42] Studerus E. Gamma A. Vollenweider F. X. (2010). Psychometric evaluation of the altered states of consciousness rating scale (OAV). PLoS ONE 5:12412. 10.1371/journal.pone.0012412 20824211 PMC2930851

[B43] Ullman J. B. Bentler P. M. (2003). Structural equation modeling. Handb. Psychol. 2003, 607–634. 10.1002/0471264385.wei0224

[B44] Vijver F. J. R. Matsumoto D. (2011). Introduction to the Methodological Issues Associated with Cross-Cultural Research. New York, NY: Cambridge University Press.

[B45] Winkelman M. J. (2005). Drug tourism or spiritual healing? Ayahuasca seekers in Amazonia. J. Psychoact. Drug. 37, 209–218. 10.1080/02791072.2005.10399803 16149335

[B46] Zinberg N. E. (1984). Drug, Set, and Setting: The Basis for Controlled Intoxicant Use. New Haven, CT: Yale University Press.

