# Supplementary material for: The Setting Questionnaire for the Ayahuasca Experience: Questionnaire Development and Internal Structure
Source: Front Psychol. 2021 Jun 23;12:679016. doi: 10.3389/fpsyg.2021.679016 (PMC8260978; doi:10.3389/fpsyg.2021.679016)

| **Supplementary material 1.** Cross-cultural English translation by two researchers, a Brazilian native Portuguese speaker and an American native English speaker. |
| --- |
| **Item** |
| L1 I entrusted all my concerns to the ritual support group. |
| L2 I felt helpless and that I had to take care of myself. ⁻ |
| L3 Those with needs were promptly taken care of. |
| L4 The ritual leadership gave me a sense of security. |
| L5 The organizers showed themselves to be inexperienced. ⁻ |
| L6 I had doubts about the organizers’ capacity to deal with possible complications. ⁻ |
| D0 The place had characteristics in common with other environments that I frequent in everyday life. # |
| D1 For my taste, the decoration was adequate. |
| D2 I would change some object or image of the decoration. ⁻ |
| D3 Certain components of the ritual didn’t align with my personal spirituality. ⁻ |
| C1 My physical position was comfortable during the ritual. |
| C2 I wish I had stayed in another position during the ritual. ⁻ |
| C3 The place where I was sitting / lying bothered me. ⁻ |
| C4 I missed having a support for my back, head or arms. ⁻ |
| I0 The ceremony was held in a sufficiently open space. # |
| I1 I felt confined. ⁻ |
| I2 I worried about the air circulation in that place. ⁻ |
| I3 I found the restroom to be inadequate. ⁻ |
| I4 There were accessible places for me to take care of my needs. |
| I5 I worried about the lack of emergency exits or other things related to safety. ⁻ |
| I6 There was a suitable place to throw up. |
| G0 Events happened that took me by surprise. ⁻ # |
| G1 The ritual took place in the manner expected. |
| G2 From start to finish, the ritual seemed under control. |
| G3 I was previously instructed about the whole ritual. |
| G4 There were times when I felt that there was a lack of instruction. ⁻ |
| S0 The other participants are similar to my friends. # |
| S00 Looking at other people bothered me. ⁻ # |
| S1 The other participants seemed to be doing well. |
| S2 I have characteristics in common with that group of people. |
| S3 I considered myself to be different from the other participants. ⁻ |
| S4 I felt that I was among peers in that group. |
| S5 Which of the following images best represents how you felt about the group during the session / ceremony? |


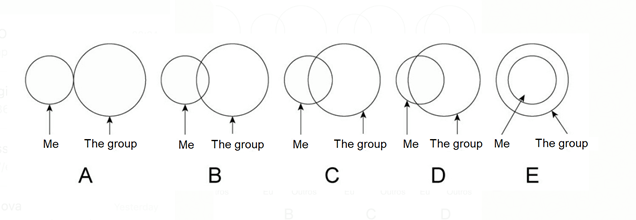

Supplement: Supplementary file 1 [file Table_1.docx]
